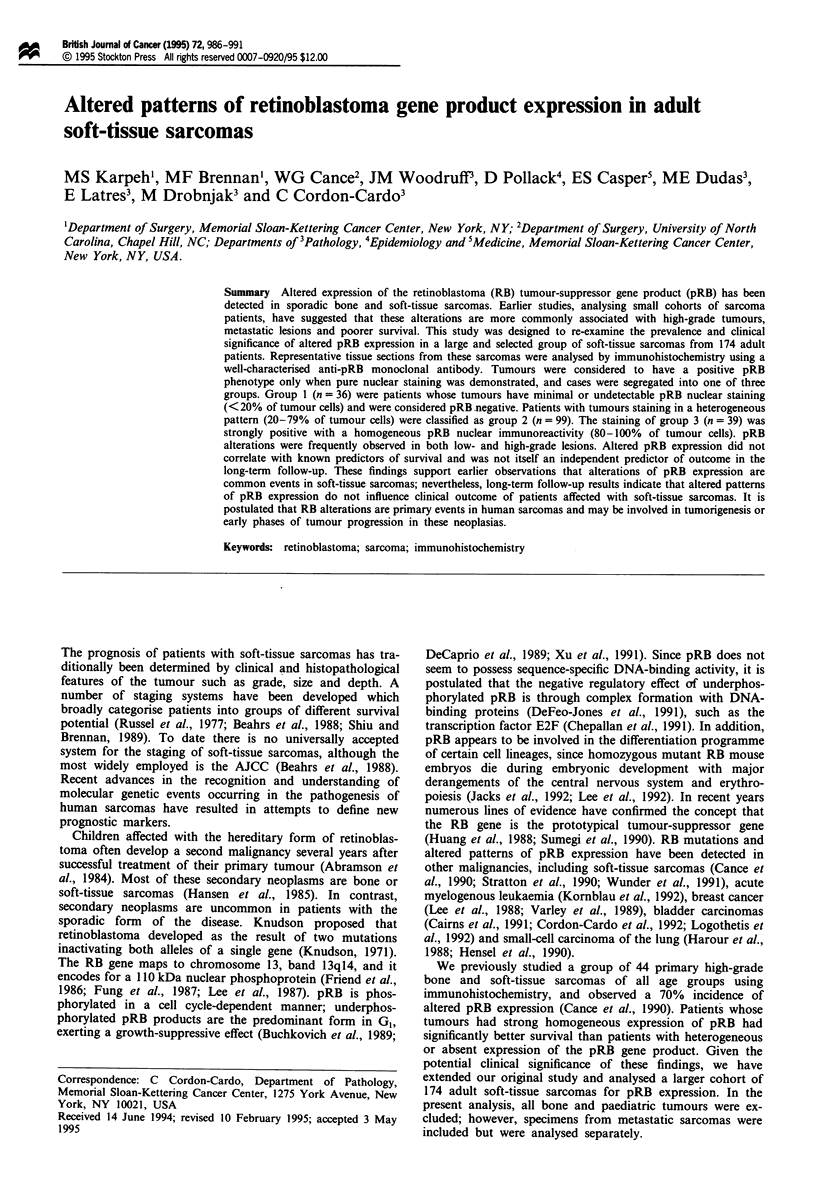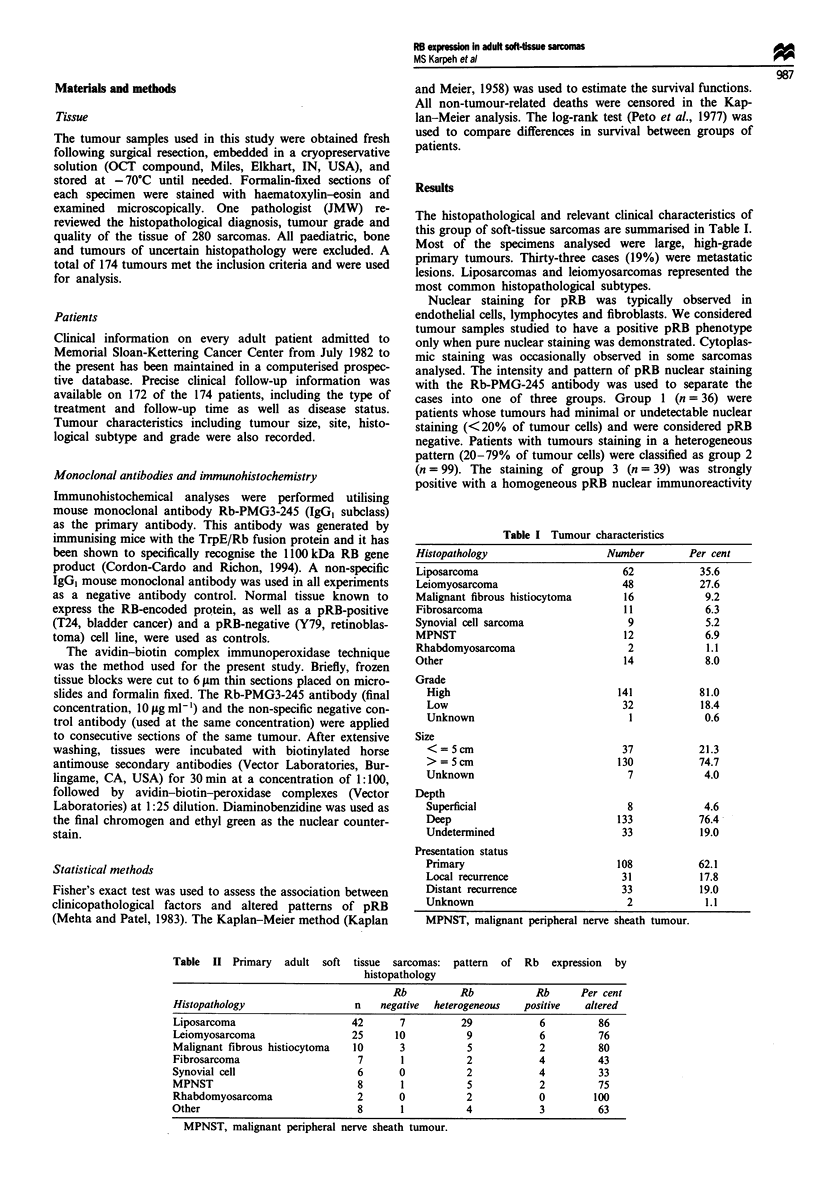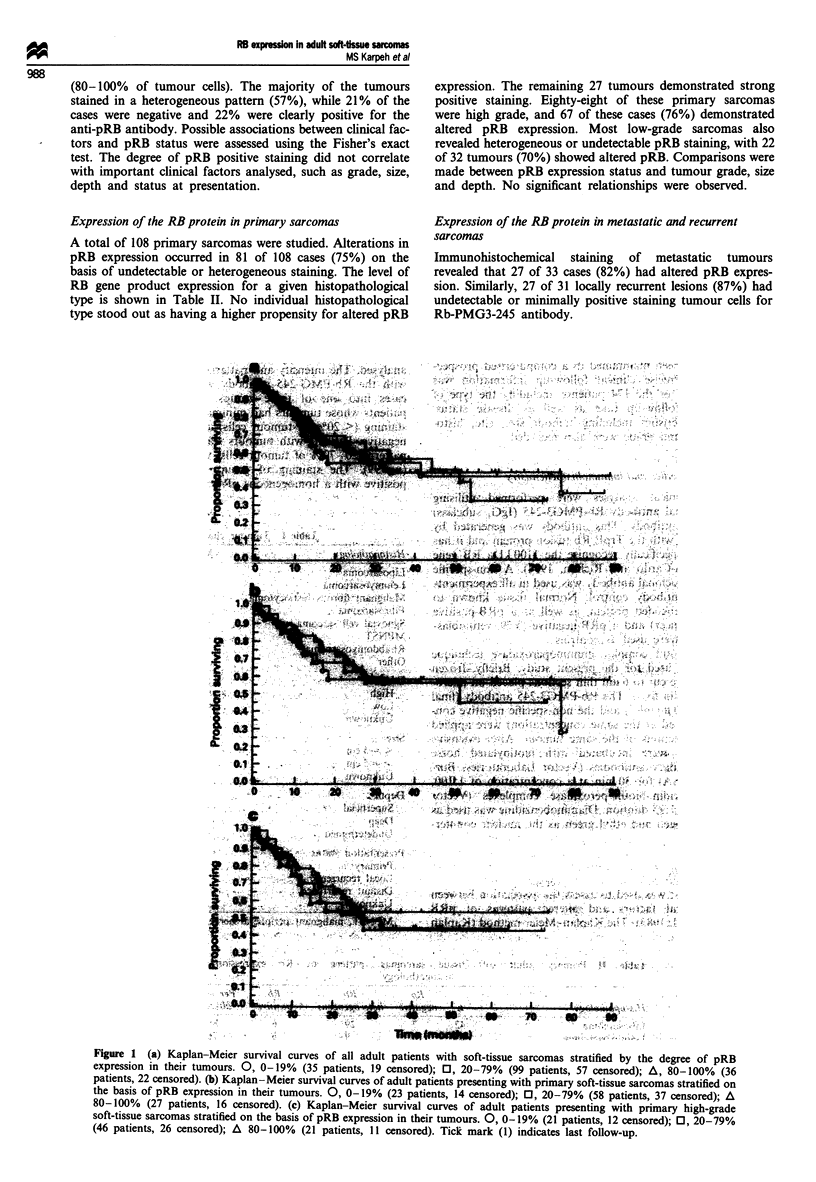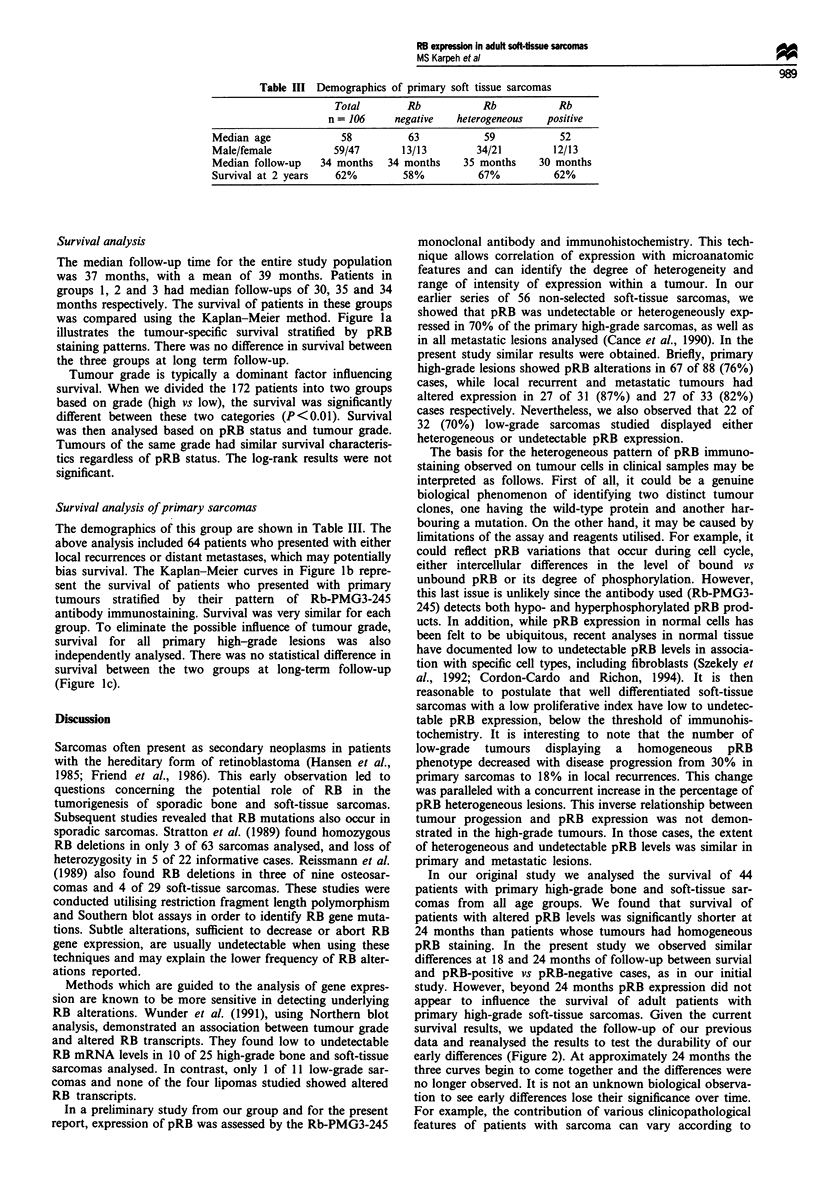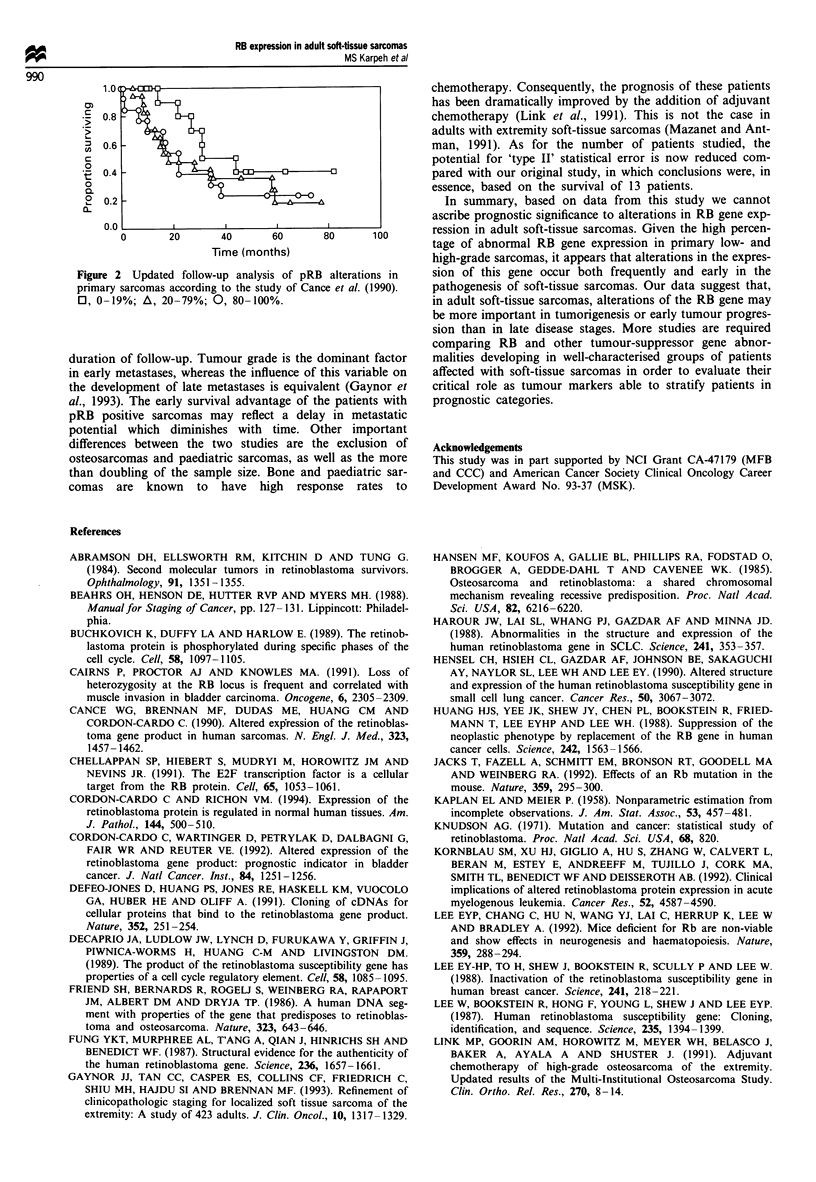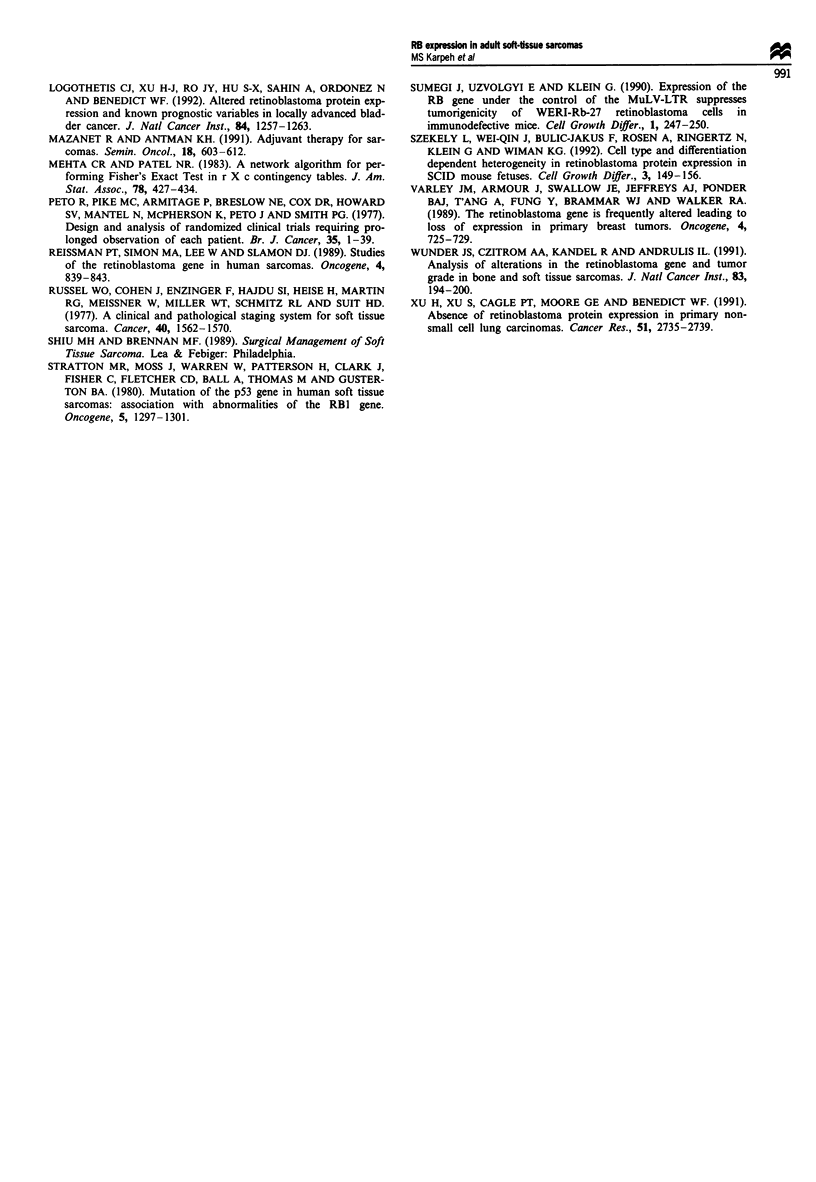# Altered patterns of retinoblastoma gene product expression in adult soft-tissue sarcomas.

**DOI:** 10.1038/bjc.1995.447

**Published:** 1995-10

**Authors:** M. S. Karpeh, M. F. Brennan, W. G. Cance, J. M. Woodruff, D. Pollack, E. S. Casper, M. E. Dudas, E. Latres, M. Drobnjak, C. Cordon-Cardo

**Affiliations:** Department of Surgery, Memorial Sloan-Kettering Cancer Center, New York, NY, USA.

## Abstract

Altered expression of the retinoblastoma (RB) tumour-suppressor gene product (pRB) has been detected in sporadic bone and soft-tissue sarcomas. Earlier studies, analysing small cohorts of sarcoma patients, have suggested that these alterations are more commonly associated with high-grade tumours, metastatic lesions and poorer survival. This study was designed to re-examine the prevalence and clinical significance of altered pRB expression in a large and selected group of soft-tissue sarcomas from 174 adult patients. Representative tissue sections from these sarcomas were analysed by immunohistochemistry using a well-characterised anti-pRB monoclonal antibody. Tumours were considered to have a positive pRB phenotype only when pure nuclear staining was demonstrated, and cases were segregated into one of three groups. Group 1 (n = 36) were patients whose tumours have minimal or undetectable pRB nuclear staining (< 20% of tumour cells) and were considered pRB negative. Patients with tumours staining in a heterogeneous pattern (20-79% of tumour cells) were classified as group 2 (n = 99). The staining of group 3 (n = 39) was strongly positive with a homogeneous pRB nuclear immunoreactivity (80-100% of tumour cells). pRB alterations were frequently observed in both low- and high-grade lesions. Altered pRB expression did not correlate with known predictors of survival and was not itself an independent predictor of outcome in the long-term follow-up. These findings support earlier observations that alterations of pRB expression are common events in soft-tissue sarcomas; nevertheless, long-term follow-up results indicate that altered patterns of pRB expression do not influence clinical outcome of patients affected with soft-tissue sarcomas. It is postulated that RB alterations are primary events in human sarcomas and may be involved in tumorigenesis or early phases of tumour progression in these neoplasias.


British Journal of Cancer (1995) 72, 986-991
© 1995 Stockton Press All rights reserved 0007-0920/95 $12.00

### Altered patterns of retinoblastoma gene product expression in adult soft-tissue sarcomas

MS Karpeh[1], MF Brennan[1], WG Cance[2], JM Woodruff[3], D Pollack[4], ES Casper[5], ME Dudas[3], E Latres[3], M Drobnjak[3] and C Cordon-Cardo[3]

[1]*Department of Surgery, Memorial Sloan-Kettering Cancer Center, New York, NY;* [2]*Department of Surgery, University of North Carolina, Chapel Hill, NC; Departments of* [3]*Pathology,* [4]*Epidemiology and* [5]*Medicine, Memorial Sloan-Kettering Cancer Center, New York, NY, USA.*

**Summary** Altered expression of the retinoblastoma (RB) tumour-suppressor gene product (pRB) has been detected in sporadic bone and soft-tissue sarcomas. Earlier studies, analysing small cohorts of sarcoma patients, have suggested that these alterations are more commonly associated with high-grade tumours, metastatic lesions and poorer survival. This study was designed to re-examine the prevalence and clinical significance of altered pRB expression in a large and selected group of soft-tissue sarcomas from 174 adult patients. Representative tissue sections from these sarcomas were analysed by immunohistochemistry using a well-characterised anti-pRB monoclonal antibody. Tumours were considered to have a positive pRB phenotype only when pure nuclear staining was demonstrated, and cases were segregated into one of three groups. Group 1 ($n = 36$) were patients whose tumours have minimal or undetectable pRB nuclear staining (<20% of tumour cells) and were considered pRB negative. Patients with tumours staining in a heterogeneous pattern (20-79% of tumour cells) were classified as group 2 ($n = 99$). The staining of group 3 ($n = 39$) was strongly positive with a homogeneous pRB nuclear immunoreactivity (80-100% of tumour cells). pRB alterations were frequently observed in both low- and high-grade lesions. Altered pRB expression did not correlate with known predictors of survival and was not itself an independent predictor of outcome in the long-term follow-up. These findings support earlier observations that alterations of pRB expression are common events in soft-tissue sarcomas; nevertheless, long-term follow-up results indicate that altered patterns of pRB expression do not influence clinical outcome of patients affected with soft-tissue sarcomas. It is postulated that RB alterations are primary events in human sarcomas and may be involved in tumorigenesis or early phases of tumour progression in these neoplasias.

**Keywords:** retinoblastoma; sarcoma; immunohistochemistry

The prognosis of patients with soft-tissue sarcomas has traditionally been determined by clinical and histopathological features of the tumour such as grade, size and depth. A number of staging systems have been developed which broadly categorise patients into groups of different survival potential (Russel *et al.*, 1977; Beahrs *et al.*, 1988; Shiu and Brennan, 1989). To date there is no universally accepted system for the staging of soft-tissue sarcomas, although the most widely employed is the AJCC (Beahrs *et al.*, 1988). Recent advances in the recognition and understanding of molecular genetic events occurring in the pathogenesis of human sarcomas have resulted in attempts to define new prognostic markers.

Children affected with the hereditary form of retinoblastoma often develop a second malignancy several years after successful treatment of their primary tumour (Abramson *et al.*, 1984). Most of these secondary neoplasms are bone or soft-tissue sarcomas (Hansen *et al.*, 1985). In contrast, secondary neoplasms are uncommon in patients with the sporadic form of the disease. Knudson proposed that retinoblastoma developed as the result of two mutations inactivating both alleles of a single gene (Knudson, 1971). The RB gene maps to chromosome 13, band 13q14, and it encodes for a 110 kDa nuclear phosphoprotein (Friend *et al.*, 1986; Fung *et al.*, 1987; Lee *et al.*, 1987). pRB is phosphorylated in a cell cycle-dependent manner; underphosphorylated pRB products are the predominant form in $G_1$, exerting a growth-suppressive effect (Buchkovich *et al.*, 1989; DeCaprio *et al.*, 1989; Xu *et al.*, 1991). Since pRB does not seem to possess sequence-specific DNA-binding activity, it is postulated that the negative regulatory effect of underphosphorylated pRB is through complex formation with DNA-binding proteins (DeFeo-Jones *et al.*, 1991), such as the transcription factor E2F (Chepallan *et al.*, 1991). In addition, pRB appears to be involved in the differentiation programme of certain cell lineages, since homozygous mutant RB mouse embryos die during embryonic development with major derangements of the central nervous system and erythropoiesis (Jacks *et al.*, 1992; Lee *et al.*, 1992). In recent years numerous lines of evidence have confirmed the concept that the RB gene is the prototypical tumour-suppressor gene (Huang *et al.*, 1988; Sumegi *et al.*, 1990). RB mutations and altered patterns of pRB expression have been detected in other malignancies, including soft-tissue sarcomas (Cance *et al.*, 1990; Stratton *et al.*, 1990; Wunder *et al.*, 1991), acute myelogenous leukaemia (Kornblau *et al.*, 1992), breast cancer (Lee *et al.*, 1988; Varley *et al.*, 1989), bladder carcinomas (Cairns *et al.*, 1991; Cordon-Cardo *et al.*, 1992; Logothetis *et al.*, 1992) and small-cell carcinoma of the lung (Harour *et al.*, 1988; Hensel *et al.*, 1990).

We previously studied a group of 44 primary high-grade bone and soft-tissue sarcomas of all age groups using immunohistochemistry, and observed a 70% incidence of altered pRB expression (Cance *et al.*, 1990). Patients whose tumours had strong homogeneous expression of pRB had significantly better survival than patients with heterogeneous or absent expression of the pRB gene product. Given the potential clinical significance of these findings, we have extended our original study and analysed a larger cohort of 174 adult soft-tissue sarcomas for pRB expression. In the present analysis, all bone and paediatric tumours were excluded; however, specimens from metastatic sarcomas were included but were analysed separately.

Correspondence: C Cordon-Cardo, Department of Pathology, Memorial Sloan-Kettering Cancer Center, 1275 York Avenue, New York, NY 10021, USA
Received 14 June 1994; revised 10 February 1995; accepted 3 May 1995

### Materials and methods

### *Tissue*

The tumour samples used in this study were obtained fresh following surgical resection, embedded in a cryopreservative solution (OCT compound, Miles, Elkhart, IN, USA), and stored at −70°C until needed. Formalin-fixed sections of each specimen were stained with haematoxylin–eosin and examined microscopically. One pathologist (JMW) re-reviewed the histopathological diagnosis, tumour grade and quality of the tissue of 280 sarcomas. All paediatric, bone and tumours of uncertain histopathology were excluded. A total of 174 tumours met the inclusion criteria and were used for analysis.

### *Patients*

Clinical information on every adult patient admitted to Memorial Sloan-Kettering Cancer Center from July 1982 to the present has been maintained in a computerised prospective database. Precise clinical follow-up information was available on 172 of the 174 patients, including the type of treatment and follow-up time as well as disease status. Tumour characteristics including tumour size, site, histological subtype and grade were also recorded.

### *Monoclonal antibodies and immunohistochemistry*

Immunohistochemical analyses were performed utilising mouse monoclonal antibody Rb-PMG3-245 ($IgG_1$ subclass) as the primary antibody. This antibody was generated by immunising mice with the TrpE/Rb fusion protein and it has been shown to specifically recognise the 1100 kDa RB gene product (Cordon-Cardo and Richon, 1994). A non-specific $IgG_1$ mouse monoclonal antibody was used in all experiments as a negative antibody control. Normal tissue known to express the RB-encoded protein, as well as a pRB-positive (T24, bladder cancer) and a pRB-negative (Y79, retinoblastoma) cell line, were used as controls.

The avidin–biotin complex immunoperoxidase technique was the method used for the present study. Briefly, frozen tissue blocks were cut to 6 μm thin sections placed on microslides and formalin fixed. The Rb-PMG3-245 antibody (final concentration, 10 $\mu g\ ml^{-1}$) and the non-specific negative control antibody (used at the same concentration) were applied to consecutive sections of the same tumour. After extensive washing, tissues were incubated with biotinylated horse antimouse secondary antibodies (Vector Laboratories, Burlingame, CA, USA) for 30 min at a concentration of 1:100, followed by avidin–biotin–peroxidase complexes (Vector Laboratories) at 1:25 dilution. Diaminobenzidine was used as the final chromogen and ethyl green as the nuclear counterstain.

### *Statistical methods*

Fisher's exact test was used to assess the association between clinicopathological factors and altered patterns of pRB (Mehta and Patel, 1983). The Kaplan–Meier method (Kaplan and Meier, 1958) was used to estimate the survival functions. All non-tumour-related deaths were censored in the Kaplan–Meier analysis. The log-rank test (Peto *et al.*, 1977) was used to compare differences in survival between groups of patients.

### Results

The histopathological and relevant clinical characteristics of this group of soft-tissue sarcomas are summarised in Table I. Most of the specimens analysed were large, high-grade primary tumours. Thirty-three cases (19%) were metastatic lesions. Liposarcomas and leiomyosarcomas represented the most common histopathological subtypes.

Nuclear staining for pRB was typically observed in endothelial cells, lymphocytes and fibroblasts. We considered tumour samples studied to have a positive pRB phenotype only when pure nuclear staining was demonstrated. Cytoplasmic staining was occasionally observed in some sarcomas analysed. The intensity and pattern of pRB nuclear staining with the Rb-PMG-245 antibody was used to separate the cases into one of three groups. Group 1 ($n = 36$) were patients whose tumours had minimal or undetectable nuclear staining (<20% of tumour cells) and were considered pRB negative. Patients with tumours staining in a heterogeneous pattern (20–79% of tumour cells) were classified as group 2 ($n = 99$). The staining of group 3 ($n = 39$) was strongly positive with a homogeneous pRB nuclear immunoreactivity

**Table I** Tumour characteristics

| *Histopathology* | *Number* | *Per cent* |
|---|---|---|
| Liposarcoma | 62 | 35.6 |
| Leiomyosarcoma | 48 | 27.6 |
| Malignant fibrous histiocytoma | 16 | 9.2 |
| Fibrosarcoma | 11 | 6.3 |
| Synovial cell sarcoma | 9 | 5.2 |
| MPNST | 12 | 6.9 |
| Rhabdomyosarcoma | 2 | 1.1 |
| Other | 14 | 8.0 |
| Grade | | |
| High | 141 | 81.0 |
| Low | 32 | 18.4 |
| Unknown | 1 | 0.6 |
| Size | | |
| < = 5 cm | 37 | 21.3 |
| > = 5 cm | 130 | 74.7 |
| Unknown | 7 | 4.0 |
| Depth | | |
| Superficial | 8 | 4.6 |
| Deep | 133 | 76.4 |
| Undetermined | 33 | 19.0 |
| Presentation status | | |
| Primary | 108 | 62.1 |
| Local recurrence | 31 | 17.8 |
| Distant recurrence | 33 | 19.0 |
| Unknown | 2 | 1.1 |

MPNST, malignant peripheral nerve sheath tumour.

**Table II** Primary adult soft tissue sarcomas: pattern of Rb expression by histopathology

| *Histopathology* | n | *Rb negative* | *Rb heterogeneous* | *Rb positive* | *Per cent altered* |
|---|---|---|---|---|---|
| Liposarcoma | 42 | 7 | 29 | 6 | 86 |
| Leiomyosarcoma | 25 | 10 | 9 | 6 | 76 |
| Malignant fibrous histiocytoma | 10 | 3 | 5 | 2 | 80 |
| Fibrosarcoma | 7 | 1 | 2 | 4 | 43 |
| Synovial cell | 6 | 0 | 2 | 4 | 33 |
| MPNST | 8 | 1 | 5 | 2 | 75 |
| Rhabdomyosarcoma | 2 | 0 | 2 | 0 | 100 |
| Other | 8 | 1 | 4 | 3 | 63 |

MPNST, malignant peripheral nerve sheath tumour.

(80–100% of tumour cells). The majority of the tumours stained in a heterogeneous pattern (57%), while 21% of the cases were negative and 22% were clearly positive for the anti-pRB antibody. Possible associations between clinical factors and pRB status were assessed using the Fisher's exact test. The degree of pRB positive staining did not correlate with important clinical factors analysed, such as grade, size, depth and status at presentation.

*Expression of the RB protein in primary sarcomas*

A total of 108 primary sarcomas were studied. Alterations in pRB expression occurred in 81 of 108 cases (75%) on the basis of undetectable or heterogeneous staining. The level of RB gene product expression for a given histopathological type is shown in Table II. No individual histopathological type stood out as having a higher propensity for altered pRB expression. The remaining 27 tumours demonstrated strong positive staining. Eighty-eight of these primary sarcomas were high grade, and 67 of these cases (76%) demonstrated altered pRB expression. Most low-grade sarcomas also revealed heterogeneous or undetectable pRB staining, with 22 of 32 tumours (70%) showed altered pRB. Comparisons were made between pRB expression status and tumour grade, size and depth. No significant relationships were observed.

*Expression of the RB protein in metastatic and recurrent sarcomas*

Immunohistochemical staining of metastatic tumours revealed that 27 of 33 cases (82%) had altered pRB expression. Similarly, 27 of 31 locally recurrent lesions (87%) had undetectable or minimally positive staining tumour cells for Rb-PMG3-245 antibody.

**Figure 1** (**a**) Kaplan–Meier survival curves of all adult patients with soft-tissue sarcomas stratified by the degree of pRB expression in their tumours. O, 0–19% (35 patients, 19 censored); □, 20–79% (99 patients, 57 censored); Δ, 80–100% (36 patients, 22 censored). (**b**) Kaplan–Meier survival curves of adult patients presenting with primary soft-tissue sarcomas stratified on the basis of pRB expression in their tumours. O, 0–19% (23 patients, 14 censored); □, 20–79% (58 patients, 37 censored); Δ 80–100% (27 patients, 16 censored). (**c**) Kaplan-Meier survival curves of adult patients presenting with primary high-grade soft-tissue sarcomas stratified on the basis of pRB expression in their tumours. O, 0–19% (21 patients, 12 censored); □, 20–79% (46 patients, 26 censored); Δ 80–100% (21 patients, 11 censored). Tick mark (1) indicates last follow-up.

Table III Demographics of primary soft tissue sarcomas

| | Total n = 106 | Rb negative | Rb heterogeneous | Rb positive |
|---|---|---|---|---|
| Median age | 58 | 63 | 59 | 52 |
| Male/female | 59/47 | 13/13 | 34/21 | 12/13 |
| Median follow-up | 34 months | 34 months | 35 months | 30 months |
| Survival at 2 years | 62% | 58% | 67% | 62% |

*Survival analysis*

The median follow-up time for the entire study population was 37 months, with a mean of 39 months. Patients in groups 1, 2 and 3 had median follow-ups of 30, 35 and 34 months respectively. The survival of patients in these groups was compared using the Kaplan-Meier method. Figure 1a illustrates the tumour-specific survival stratified by pRB staining patterns. There was no difference in survival between the three groups at long term follow-up.

Tumour grade is typically a dominant factor influencing survival. When we divided the 172 patients into two groups based on grade (high *vs* low), the survival was significantly different between these two categories ($P<0.01$). Survival was then analysed based on pRB status and tumour grade. Tumours of the same grade had similar survival characteristics regardless of pRB status. The log-rank results were not significant.

*Survival analysis of primary sarcomas*

The demographics of this group are shown in Table III. The above analysis included 64 patients who presented with either local recurrences or distant metastases, which may potentially bias survival. The Kaplan-Meier curves in Figure 1b represent the survival of patients who presented with primary tumours stratified by their pattern of Rb-PMG3-245 antibody immunostaining. Survival was very similar for each group. To eliminate the possible influence of tumour grade, survival for all primary high-grade lesions was also independently analysed. There was no statistical difference in survival between the two groups at long-term follow-up (Figure 1c).

### Discussion

Sarcomas often present as secondary neoplasms in patients with the hereditary form of retinoblastoma (Hansen *et al.*, 1985; Friend *et al.*, 1986). This early observation led to questions concerning the potential role of RB in the tumorigenesis of sporadic bone and soft-tissue sarcomas. Subsequent studies revealed that RB mutations also occur in sporadic sarcomas. Stratton *et al.* (1989) found homozygous RB deletions in only 3 of 63 sarcomas analysed, and loss of heterozygosity in 5 of 22 informative cases. Reissmann *et al.* (1989) also found RB deletions in three of nine osteosarcomas and 4 of 29 soft-tissue sarcomas. These studies were conducted utilising restriction fragment length polymorphism and Southern blot assays in order to identify RB gene mutations. Subtle alterations, sufficient to decrease or abort RB gene expression, are usually undetectable when using these techniques and may explain the lower frequency of RB alterations reported.

Methods which are guided to the analysis of gene expression are known to be more sensitive in detecting underlying RB alterations. Wunder *et al.* (1991), using Northern blot analysis, demonstrated an association between tumour grade and altered RB transcripts. They found low to undetectable RB mRNA levels in 10 of 25 high-grade bone and soft-tissue sarcomas analysed. In contrast, only 1 of 11 low-grade sarcomas and none of the four lipomas studied showed altered RB transcripts.

In a preliminary study from our group and for the present report, expression of pRB was assessed by the Rb-PMG3-245 monoclonal antibody and immunohistochemistry. This technique allows correlation of expression with microanatomic features and can identify the degree of heterogeneity and range of intensity of expression within a tumour. In our earlier series of 56 non-selected soft-tissue sarcomas, we showed that pRB was undetectable or heterogeneously expressed in 70% of the primary high-grade sarcomas, as well as in all metastatic lesions analysed (Cance *et al.*, 1990). In the present study similar results were obtained. Briefly, primary high-grade lesions showed pRB alterations in 67 of 88 (76%) cases, while local recurrent and metastatic tumours had altered expression in 27 of 31 (87%) and 27 of 33 (82%) cases respectively. Nevertheless, we also observed that 22 of 32 (70%) low-grade sarcomas studied displayed either heterogeneous or undetectable pRB expression.

The basis for the heterogeneous pattern of pRB immunostaining observed on tumour cells in clinical samples may be interpreted as follows. First of all, it could be a genuine biological phenomenon of identifying two distinct tumour clones, one having the wild-type protein and another harbouring a mutation. On the other hand, it may be caused by limitations of the assay and reagents utilised. For example, it could reflect pRB variations that occur during cell cycle, either intercellular differences in the level of bound *vs* unbound pRB or its degree of phosphorylation. However, this last issue is unlikely since the antibody used (Rb-PMG3-245) detects both hypo- and hyperphosphorylated pRB products. In addition, while pRB expression in normal cells has been felt to be ubiquitous, recent analyses in normal tissue have documented low to undetectable pRB levels in association with specific cell types, including fibroblasts (Szekely *et al.*, 1992; Cordon-Cardo and Richon, 1994). It is then reasonable to postulate that well differentiated soft-tissue sarcomas with a low proliferative index have low to undetectable pRB expression, below the threshold of immunohistochemistry. It is interesting to note that the number of low-grade tumours displaying a homogeneous pRB phenotype decreased with disease progression from 30% in primary sarcomas to 18% in local recurrences. This change was paralleled with a concurrent increase in the percentage of pRB heterogeneous lesions. This inverse relationship between tumour progession and pRB expression was not demonstrated in the high-grade tumours. In those cases, the extent of heterogeneous and undetectable pRB levels was similar in primary and metastatic lesions.

In our original study we analysed the survival of 44 patients with primary high-grade bone and soft-tissue sarcomas from all age groups. We found that survival of patients with altered pRB levels was significantly shorter at 24 months than patients whose tumours had homogeneous pRB staining. In the present study we observed similar differences at 18 and 24 months of follow-up between survial and pRB-positive *vs* pRB-negative cases, as in our initial study. However, beyond 24 months pRB expression did not appear to influence the survival of adult patients with primary high-grade soft-tissue sarcomas. Given the current survival results, we updated the follow-up of our previous data and reanalysed the results to test the durability of our early differences (Figure 2). At approximately 24 months the three curves begin to come together and the differences were no longer observed. It is not an unknown biological observation to see early differences lose their significance over time. For example, the contribution of various clinicopathological features of patients with sarcoma can vary according to

**Figure 2** Updated follow-up analysis of pRB alterations in primary sarcomas according to the study of Cance *et al.* (1990). □, 0–19%; △, 20–79%; ○, 80–100%.

duration of follow-up. Tumour grade is the dominant factor in early metastases, whereas the influence of this variable on the development of late metastases is equivalent (Gaynor *et al.*, 1993). The early survival advantage of the patients with pRB positive sarcomas may reflect a delay in metastatic potential which diminishes with time. Other important differences between the two studies are the exclusion of osteosarcomas and paediatric sarcomas, as well as the more than doubling of the sample size. Bone and paediatric sarcomas are known to have high response rates to chemotherapy. Consequently, the prognosis of these patients has been dramatically improved by the addition of adjuvant chemotherapy (Link *et al.*, 1991). This is not the case in adults with extremity soft-tissue sarcomas (Mazanet and Antman, 1991). As for the number of patients studied, the potential for 'type II' statistical error is now reduced compared with our original study, in which conclusions were, in essence, based on the survival of 13 patients.

In summary, based on data from this study we cannot ascribe prognostic significance to alterations in RB gene expression in adult soft-tissue sarcomas. Given the high percentage of abnormal RB gene expression in primary low- and high-grade sarcomas, it appears that alterations in the expression of this gene occur both frequently and early in the pathogenesis of soft-tissue sarcomas. Our data suggest that, in adult soft-tissue sarcomas, alterations of the RB gene may be more important in tumorigenesis or early tumour progression than in late disease stages. More studies are required comparing RB and other tumour-suppressor gene abnormalities developing in well-characterised groups of patients affected with soft-tissue sarcomas in order to evaluate their critical role as tumour markers able to stratify patients in prognostic categories.

**Acknowledgements**
This study was in part supported by NCI Grant CA-47179 (MFB and CCC) and American Cancer Society Clinical Oncology Career Development Award No. 93-37 (MSK).

**References**

ABRAMSON DH, ELLSWORTH RM, KITCHIN D AND TUNG G. (1984). Second molecular tumors in retinoblastoma survivors. *Ophthalmology*, **91**, 1351–1355.

BEAHRS OH, HENSON DE, HUTTER RVP AND MYERS MH. (1988). *Manual for Staging of Cancer*, pp. 127–131. Lippincott: Philadelphia.

BUCHKOVICH K, DUFFY LA AND HARLOW E. (1989). The retinoblastoma protein is phosphorylated during specific phases of the cell cycle. *Cell*, **58**, 1097–1105.

CAIRNS P, PROCTOR AJ AND KNOWLES MA. (1991). Loss of heterozygosity at the RB locus is frequent and correlated with muscle invasion in bladder carcinoma. *Oncogene*, **6**, 2305–2309.

CANCE WG, BRENNAN MF, DUDAS ME, HUANG CM AND CORDON-CARDO C. (1990). Altered expression of the retinoblastoma gene product in human sarcomas. *N. Engl. J. Med.*, **323**, 1457–1462.

CHELLAPPAN SP, HIEBERT S, MUDRYI M, HOROWITZ JM AND NEVINS JR. (1991). The E2F transcription factor is a cellular target from the RB protein. *Cell*, **65**, 1053–1061.

CORDON-CARDO C AND RICHON VM. (1994). Expression of the retinoblastoma protein is regulated in normal human tissues. *Am. J. Pathol.*, **144**, 500–510.

CORDON-CARDO C, WARTINGER D, PETRYLAK D, DALBAGNI G, FAIR WR AND REUTER VE. (1992). Altered expression of the retinoblastoma gene product: prognostic indicator in bladder cancer. *J. Natl Cancer. Inst.*, **84**, 1251–1256.

DEFEO-JONES D, HUANG PS, JONES RE, HASKELL KM, VUOCOLO GA, HUBER HE AND OLIFF A. (1991). Cloning of cDNAs for cellular proteins that bind to the retinoblastoma gene product. *Nature*, **352**, 251–254.

DECAPRIO JA, LUDLOW JW, LYNCH D, FURUKAWA Y, GRIFFIN J, PIWNICA-WORMS H, HUANG C-M AND LIVINGSTON DM. (1989). The product of the retinoblastoma susceptibility gene has properties of a cell cycle regulatory element. *Cell*, **58**, 1085–1095.

FRIEND SH, BERNARDS R, ROGELJ S, WEINBERG RA, RAPAPORT JM, ALBERT DM AND DRYJA TP. (1986). A human DNA segment with properties of the gene that predisposes to retinoblastoma and osteosarcoma. *Nature*, **323**, 643–646.

FUNG YKT, MURPHREE AL, T'ANG A, QIAN J, HINRICHS SH AND BENEDICT WF. (1987). Structural evidence for the authenticity of the human retinoblastoma gene. *Science*, **236**, 1657–1661.

GAYNOR JJ, TAN CC, CASPER ES, COLLINS CF, FRIEDRICH C, SHIU MH, HAJDU SI AND BRENNAN MF. (1993). Refinement of clinicopathologic staging for localized soft tissue sarcoma of the extremity: A study of 423 adults. *J. Clin. Oncol.*, **10**, 1317–1329.

HANSEN MF, KOUFOS A, GALLIE BL, PHILLIPS RA, FODSTAD O, BROGGER A, GEDDE-DAHL T AND CAVENEE WK. (1985). Osteosarcoma and retinoblastoma: a shared chromosomal mechanism revealing recessive predisposition. *Proc. Natl Acad. Sci. USA*, **82**, 6216–6220.

HAROUR JW, LAI SL, WHANG PJ, GAZDAR AF AND MINNA JD. (1988). Abnormalities in the structure and expression of the human retinoblastoma gene in SCLC. *Science*, **241**, 353–357.

HENSEL CH, HSIEH CL, GAZDAR AF, JOHNSON BE, SAKAGUCHI AY, NAYLOR SL, LEE WH AND LEE EY. (1990). Altered structure and expression of the human retinoblastoma susceptibility gene in small cell lung cancer. *Cancer Res.*, **50**, 3067–3072.

HUANG HJS, YEE JK, SHEW JY, CHEN PL, BOOKSTEIN R, FRIEDMANN T, LEE EYHP AND LEE WH. (1988). Suppression of the neoplastic phenotype by replacement of the RB gene in human cancer cells. *Science*, **242**, 1563–1566.

JACKS T, FAZELL A, SCHMITT EM, BRONSON RT, GOODELL MA AND WEINBERG RA. (1992). Effects of an Rb mutation in the mouse. *Nature*, **359**, 295–300.

KAPLAN EL AND MEIER P. (1958). Nonparametric estimation from incomplete observations. *J. Am. Stat. Assoc.*, **53**, 457–481.

KNUDSON AG. (1971). Mutation and cancer: statistical study of retinoblastoma. *Proc. Natl Acad. Sci. USA*, **68**, 820.

KORNBLAU SM, XU HJ, GIGLIO A, HU S, ZHANG W, CALVERT L, BERAN M, ESTEY E, ANDREEFF M, TUJILLO J, CORK MA, SMITH TL, BENEDICT WF AND DEISSEROTH AB. (1992). Clinical implications of altered retinoblastoma protein expression in acute myelogenous leukemia. *Cancer Res.*, **52**, 4587–4590.

LEE EYP, CHANG C, HU N, WANG YJ, LAI C, HERRUP K, LEE W AND BRADLEY A. (1992). Mice deficient for Rb are non-viable and show effects in neurogenesis and haematopoiesis. *Nature*, **359**, 288–294.

LEE EY-HP, TO H, SHEW J, BOOKSTEIN R, SCULLY P AND LEE W. (1988). Inactivation of the retinoblastoma susceptibility gene in human breast cancer. *Science*, **241**, 218–221.

LEE W, BOOKSTEIN R, HONG F, YOUNG L, SHEW J AND LEE EYP. (1987). Human retinoblastoma susceptibility gene: Cloning, identification, and sequence. *Science*, **235**, 1394–1399.

LINK MP, GOORIN AM, HOROWITZ M, MEYER WH, BELASCO J, BAKER A, AYALA A AND SHUSTER J. (1991). Adjuvant chemotherapy of high-grade osteosarcoma of the extremity. Updated results of the Multi-Institutional Osteosarcoma Study. *Clin. Ortho. Rel. Res.*, **270**, 8–14.

LOGOTHETIS CJ, XU H-J, RO JY, HU S-X, SAHIN A, ORDONEZ N AND BENEDICT WF. (1992). Altered retinoblastoma protein expression and known prognostic variables in locally advanced bladder cancer. *J. Natl Cancer Inst.*, **84**, 1257–1263.

MAZANET R AND ANTMAN KH. (1991). Adjuvant therapy for sarcomas. *Semin. Oncol.*, **18**, 603–612.

MEHTA CR AND PATEL NR. (1983). A network algorithm for performing Fisher's Exact Test in r X c contingency tables. *J. Am. Stat. Assoc.*, **78**, 427–434.

PETO R, PIKE MC, ARMITAGE P, BRESLOW NE, COX DR, HOWARD SV, MANTEL N, MCPHERSON K, PETO J AND SMITH PG. (1977). Design and analysis of randomized clinical trials requiring prolonged observation of each patient. *Br. J. Cancer*, **35**, 1–39.

REISSMAN PT, SIMON MA, LEE W AND SLAMON DJ. (1989). Studies of the retinoblastoma gene in human sarcomas. *Oncogene*, **4**, 839–843.

RUSSEL WO, COHEN J, ENZINGER F, HAJDU SI, HEISE H, MARTIN RG, MEISSNER W, MILLER WT, SCHMITZ RL AND SUIT HD. (1977). A clinical and pathological staging system for soft tissue sarcoma. *Cancer*, **40**, 1562–1570.

SHIU MH AND BRENNAN MF. (1989). *Surgical Management of Soft Tissue Sarcoma*. Lea & Febiger: Philadelphia.

STRATTON MR, MOSS J, WARREN W, PATTERSON H, CLARK J, FISHER C, FLETCHER CD, BALL A, THOMAS M AND GUSTERTON BA. (1980). Mutation of the p53 gene in human soft tissue sarcomas: association with abnormalities of the RB1 gene. *Oncogene*, **5**, 1297–1301.

SUMEGI J, UZVOLGYI E AND KLEIN G. (1990). Expression of the RB gene under the control of the MuLV-LTR suppresses tumorigenicity of WERI-Rb-27 retinoblastoma cells in immunodefective mice. *Cell Growth Differ.*, **1**, 247–250.

SZEKELY L, WEI-QIN J, BULIC-JAKUS F, ROSEN A, RINGERTZ N, KLEIN G AND WIMAN KG. (1992). Cell type and differentiation dependent heterogeneity in retinoblastoma protein expression in SCID mouse fetuses. *Cell Growth Differ.*, **3**, 149–156.

VARLEY JM, ARMOUR J, SWALLOW JE, JEFFREYS AJ, PONDER BAJ, T'ANG A, FUNG Y, BRAMMAR WJ AND WALKER RA. (1989). The retinoblastoma gene is frequently altered leading to loss of expression in primary breast tumors. *Oncogene*, **4**, 725–729.

WUNDER JS, CZITROM AA, KANDEL R AND ANDRULIS IL. (1991). Analysis of alterations in the retinoblastoma gene and tumor grade in bone and soft tissue sarcomas. *J. Natl Cancer Inst.*, **83**, 194–200.

XU H, XU S, CAGLE PT, MOORE GE AND BENEDICT WF. (1991). Absence of retinoblastoma protein expression in primary non-small cell lung carcinomas. *Cancer Res.*, **51**, 2735–2739.